# Florfenicol induces early embryonic death in eggs collected from treated hens

**DOI:** 10.1186/s12917-015-0536-0

**Published:** 2015-08-18

**Authors:** S. AL-Shahrani, V. Naidoo

**Affiliations:** Department of Paraclinical Sciences, Faculty of Veterinary Science, University of Pretoria, Private Bag X04, Onderstepoort, 0110 South Africa

**Keywords:** Florfenicol, Broiler breeders, Chickens, Embryonic toxicity, Hatchability

## Abstract

**Background:**

Florfenicol, a commonly used veterinary antibiotic, was reported to have caused a severe drop in egg hatchability following its off-label use on a broiler breeder farm in South Africa. According to the pharmacovigilance report, hatchability dropped by 80 % for up to a week following a five day course at 10 mg/kg (both males and females treated metaphylactically) to manage an *Escherichia coli* infection. While mammalian toxicity studies indicate the potential for early embryonic death in utero or testicular damage, no literature is available on the avian toxicity of florfenicol. For this study we investigated the effects of florfenicol at various doses from 10 to 90 mg/kg on the egg hatchability in a breeder flock we kept and established under controlled conditions, with the same cockerels and hens being exposed in a phased manner.

**Results:**

Following five days of oral exposure, no toxic signs were evident in any of the cockerels or hens treated at doses up to 90 mg/kg. Treatment of only the cockerels had no effect on egg hatchability, while treatment of only the hens at doses of 60 and 90 mg/kg resulted in decreased hatchability of 0 % in comparison to 70 % of the control as early 24 h after treatment. In all cases, decreased hatchability was associated with embryonic death at 5 days of development. The toxic effects of florfenicol were completely reversible with comparable hatchability being present by day 4 post-treatment withdrawal. Toxicity correlated with total egg florfenicol concentrations with an LC_50_ of 1.07 μg/g.

**Conclusion:**

Florfenicol appears to be toxic to the developing chick embryo at around day 5 of incubation, in the absence of related toxicity in the hen or cockerel.

## Background

A spontaneous adverse reaction was reported to the Faculty of Veterinary Science, University of Pretoria, South Africa (reporter’s details are kept confidential), describing an outbreak of *Escherichia coli* induced mortalities in a grandparent layer breeder flock in South Africa following the use of florfenicol. The report was according to South African legislation which supports the reporting of adverse reactions even when the arising through the use of non-registered and compounded medicines [1]. According to the spontaneous report, sensitivity testing showed florfenicol as the only drug with good efficacy. Florfenicol is a fluorinated derivative of cloramphenicol [2], with the chemical name 2,2-dichloro-N-[1-(fluoromethyl)-2-hydroxy-2-[(methylsulfonyl) phenyl] ethyl] acetamide within the amphenicol group of antibiotics, with wide scale use in the treatment of pneumonia in food-producing animals [3]. In contrast to chloramphenicol which has been associated with the occurrence of aplastic anaemia in people, florfenicol does not contain the nitro group making it safe for use in food-producing animals [4]. Florfenicol is primarily bacteriostatic, with a range of activity against many Gram-negative and Gram-positive organisms, including certain chloramphenicol-resistant strains of *Escherichia coli*, *Salmonella typhimurium* and *Staphylococcus aureus* [5]. Florfenicol functions through the suppression of protein synthesis by inhibiting the peptidyl transferase enzyme as well as ribosomal translocation [6, 7].

While not registered for use in breeder fowls, the treating veterinarian made an informed decision to treat the birds with the non-registered florfenicol. The birds were subsequently treated with a compounded florfenicol formulation (active in glycerol), produced by a local pharmacy (Vtech compounding solutions), in the water at an equivalent dose of 10 mg/kg for 5 days. According to the spontaneous report the hens made a complete recovery with no further mortalities being recorded. A major side effect of reduced hatchability of 80 % was reported, which lasted for a week from treatment initiation before returning to normal. Despite numerous attempts, further information was not forthcoming from the veterinarian or the farm in question.

While florfenicol is a described reproductive toxin this information has largely been obtained from classical mammalian laboratory toxicity tests. According to the summary material safety data sheet published by Schering-Plough [8], an oral two-generation reproductive study in rats (12 mg/kg/day for 90 days) revealed reduced epididymal weights, decreased pup survival, and reduced milk production. Another two-generational teratogenicity study in mice, florfenicol (40, 120, or 400 mg/kg by gavage) on days 6-15 of gestation showed signs of embryo lethality at the high dose. With an extensive literature review yielding no further information on the *in vivo* avian toxic potential of florfenicol, the aim of the following study was to establish the toxic effects of florfenicol on egg hatchability in a commercially representative breeding flock under controlled conditions.

## Methods

### Animals

Hyline hens were purchased from Eggspert eggs (Kempton Park, South Africa) and cockerels from HyLine SA (Midrand-South Africa) at the age of 15 weeks and already vaccinated for local conditions. The birds were individually marked with patagial tags prior to being randomly divided into four groups of 30 hens and 4 cockerels. The hen to cockerel ratio was at the recommended ratio of 8:1 for highest fertility within breeder flocks [9]. Birds were housed in large open pens of which the floors were covered with wood shavings in naturally ventilated rooms with curtained windows and had access to a standard diet fortified with salinomycin at 50 ppm (Avi-Products Pty) and potable municipal water *ad libitum*. Research was approved by the Animal Use and Care Committee of the University of Pretoria (V070-08) and was conducted according the South Africa standards for the use of animals in research.

### Experimental design

Florfenicol was supplied by Vtech Compounding Solutions Pharmacy (Midrand, South Africa) as the exact formulation (florfenicol in glycerol to a final concentration of 30 mg/ml) reported in the spontaneous report. The study was completed in three phases with the same group of birds being used in all phases and a wash-out period of 3 weeks between phases. Treatments were administered once daily for 5 days by direct administration into the crop to ensure the correct exposure. In phase 1, only the hens were treated at concentrations of 10, 20 and 30 mg/kg or sterile water. In phase 2, only the cockerels were treated with florfenicol at 30, 60, and 90 mg/kg or sterile water (Higher doses were selected due to the absence of toxicity in phase 1). In phase 3, only the hens were treated with florfenicol at 30, 60 and 90 mg/kg or sterile water. Prior to each phase the birds were all deemed to be clinically healthy, within normal weight limits, with all treatment groups having equivalent or better egg hatchability to the control.

### Monitoring of birds and chicks

Hens and cockerels were evaluated daily for clinical signs of toxicity. Eggs were also collected on selected days for incubation (Buckeye egg incubator at 37 to 37.5 °C at 50 % relative humidity, automatically turned hourly) to determine fertility by candling, egg break-outs or hatchings. A staggered pattern was adopted due to limited incubator space (Table 1). In total 30 eggs were incubated per time point for phase 1 and 2 and the majority of phase 3 (Tables 1, 2, 3). Fewer eggs were available for incubation in phase 3 due to a sequential termination of the adult hens for necropsy evaluation. Final egg evaluations by candling was undertaken on day 18. Eggs deemed infertile on candling were subjected to break-outs to determine time of embryonic death according to standard charts [10], while fertile eggs were taken to hatch. Fertility from the break-outs is presented as the percentage of eggs with viable foetuses (% fertility). For eggs taken to hatch, chicks were evaluated for their general quality; such as ability to stand, feather cover, shape of their beaks, movement ability and presence or absence of open navels. At the end of phase 3, hens in groups of five were sacrificed using CO_2_ at 0, 1, 2, 3 and 4 days post drug withdrawal for general necropsy evaluation.

**Table 1:** Percentage fertility recorded during the study when only the hens were treated at the 10, 20 or 30 mg/kg (*n* = 30) and the method of evaluation

Time	Event	Dose administered (mg/kg)				Method
		0	10	20	30	
−24	Day before treatment	88	93	87	77	Hatch
24	24 h after 1’st Treatment	89	78	76	76	Break-out
72	24 h after third treatment	74	70	73	61	Hatch
96	24 h after 4’th treatment	86	90	73	74	Break-out
120	Day 1 after withdrawal	94	85	79	85	Break-out
168	Day 3 after withdrawal	75	73	66	78	Hatch
192	Day 4 after withdrawal	81	82	70	89	Break-out
240	Day 6 after withdrawal	83	82	90	87	Break-out
264	Day 7 after withdrawal	77	77	89	78	Hatch

Time- indicates the time of eggs collection in relation to treatment. Method- indicates the methodology used to ascertain fertility for the specified time point. Values are presented as the percentage fertility of the total number of eggs incubated

### Florfenicol quantification in the egg

**Fig. 1:**
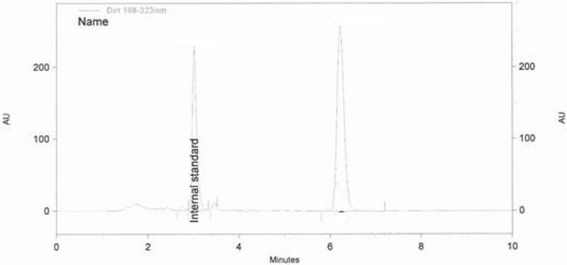
Chromatogram for the 10 μg/ml sample, with the thiamphenicol internal standard

In phase 3, eggs collected on days 1 to 5 after treatment withdrawal were randomly collected from each group for quantification of their florfenicol concentrations using a modified method of Varma [3]. Eggs were cracked, shells discarded and the yolk/albumin homogenised. Two grams of egg homogenate in natural proportions were mixed with 100 μl of 10 μg/ml thiamphenicol (internal standard) and 9 ml ethyl acetate, vortexed and subsequently centrifuged at 2000 × *g* for 15 min. The resulting supernatant was decanted into a new tube, dried off for 30 min at 60 °C under a stream of nitrogen, prior to being mixed with 2 ml high pure water (MilliQ) and 2 ml hexane and re-centrifuged at 2000 × *g* for 15 min. For final extraction the supernatant was subjected to solid phase extraction (Varian BondElut C18) on cartridges primed with 4 ml methanol, followed by 4 ml MilliQ50 water. After the sample loading, a second wash was performed using 2 ml MilliQ50 water after which the cartridge was allowed to dry under vacuum for 5 min. Final elution was with 3 ml methanol under vacuum for 5 min. The eluent was dried under a stream of nitrogen for 30 min at 60 °C, prior to being reconstituted in 500ul of 30 % acetonitrile in reverse osmosis water (mobile phase) of which 100 μl was injected onto the column [Phenomenex guard cartridges (AJO-4287) and LG reverse phase, Luna 5μaC18 (2); 100A; 150 × 4.6 mm] under isocratic flow of 1 ml/min. Detection was via diode array on a Beckman HPLC at 223 nm (Fig. 1).

**Fig. 2:**
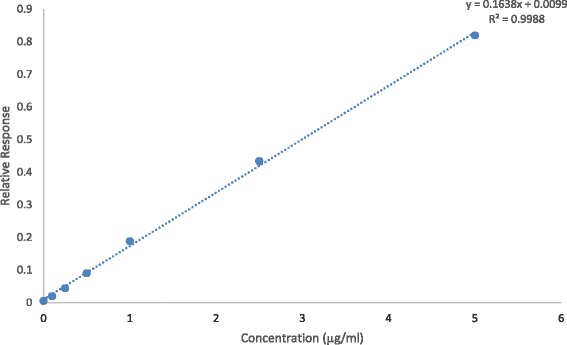
Fortified standard curve for the various concentrations of florfenicol spiked into egg samples

Results were interpreted against a fortified curve in which known concentrations of florfenicol and thiamphenicol were added to eggs collected from the control birds for which were maintained strictly florfenicol free for the entire study (Fig. 2). The method was linear from 0.1 to 10 μg/g, with 0.1 μg/ml being the visual limit of the detection. The intra-day recovery (*n* = 2) was 91.96 and 103.02 % for the lowest and highest concentration with a corresponding relative standard deviation (RSD) of 3.8 and 1.21 % respectively. The inter-day recovery (*n* = 5) was 103.71 and 104.81 % for the lowest and highest concentration, with the corresponding RSD being 9.23 and 5.56 % respectively. All analysis was undertaken over a period of 5 days.

Florfenicol concentrations are presented for the different dose groups as mean and standard deviation. Egg concentrations were compared to the corresponding percentage fertility obtained by egg break-out. The florfenicol concentrations were plotted against percentage fertility on the natural logarithm scale to ascertain if a linear concentration versus response was present. The estimated concentration which could induce a 50 % reduction in fertility (LC50 %) was calculated from the best fit equation obtained by linear regression.

## Results

For this study we investigated a pharmacovigilance report suggesting the reproductive toxic effect of florfenicol in a commercial breeding chicken flock. To obtain realistic results, we established breeding flocks with a hen to cockerel ratio of 8:1 as seen on commercial farms. To ascertain whether the effect was on the hens in the flocks, the cockerels in the flocks or just on eggs, the study was divided into three phases. For all three phase the same breeding birds were used. No clinical signs of toxicity were evident in any bird during the study.

The fertility of the flock was generally excellent and was above 70 % at the start of each phase as determined by break-outs, and was comparable to the industry standard [11] with hatchability decreasing, as expected with the flock aging. Fertility as measured by hatchings were not as favourable as for the break-outs on certain days. However, since the fluctuations are a natural occurrence, the results for the treatment groups were always compared to the control group to rule out incidental environmental influences on hatchability.

In phase 1 (low dose hen) the only abnormality was a mild drop in fertility immediately after the fourth treatment of 72 and 74 % for the 20 and 30 mg/kg groups respectively in comparison to 93 % for the control group on break-outs (Table 1). This decrease corresponded with similar decrease in chicks hatching. No other time points in phase 1 showed signs of toxicity. In phase 2 (high dose males), no changes in fertility were observed for any of the groups (*n* = 30) with all groups showing similar % fertility to the control group (Table 2). No abnormalities were seen in any of the hatched chicks in phase 1 and 2.

**Table 2:** Percentage fertility recorded during the study when only the cockerels were treated at the 30, 60 or 90 mg/kg (*n* = 30) and the method of evaluation

Time	Event	Dose administered (mg/kg)				Method
		0	30	60	90	
−24	Day before treatment	81	72	71	67	Hatch
24	24 h after 1’st Treatment	92	92	88	92	Break-out
72	24 h after third treatment	75	92	73	76	Hatch
96	24 h after 4’th treatment	87	86	83	77	Break-out
120	Day 1 after withdrawal	70	77	83	83	Break-out
168	Day 3 after withdrawal	70	65	62	77	Hatch
192	Day 4 after withdrawal	87	92	87	87	Break-out
240	Day 6 after withdrawal	86	90	93	92	Break-out
264	Day 7 after withdrawal	63	77	63	57	Hatch

Time- indicates the time of eggs collection in relation to treatment. Method- indicates the methodology used to ascertain fertility for the specified time point. Values are presented as the percentage fertility of the total number of eggs incubated

In phase 3, evidence of decreased fertility was present in both the 60 and 90 mg/kg groups (Table 3), with the day 18 percentage fertility being 3 and 0 % respectively as early as 24 h after the first dose (*n* = 30). In contrast the control group had a percentage fertility of 70 %. Fertility remained low until day 4 after treatment cessation (*n* = 5), with no chicks hatching from these treatment groups. Hereafter hatchability returned to control levels with no signs of abnormalities being present in hatching chicks. While none of the hens showed any signs of abnormalities on necropsy, egg breakouts showed that foetal deaths resulted at approximately day 5 of development.

**Table 3:** Percentage fertility recorded during the study when only the hens were treated at the 30, 60 or 90 mg/kg and the method of evaluation

Time	Event	n	Dose administered (mg/kg)				Method
			0	30	60	90	
−24	Day before treatment	30	70	77	90	73	Hatch
24	24 h after 1’st Treatment	30	70	69	3	0	Break-out
72	24 h after third treatment	30	50	52	0	0	Hatch
96	24 h after 4’th treatment	30	71	81	0	0	Break-out
120	Day 1 after withdrawal	25	71	64	12	8	Break-out
168	Day 3 after withdrawal	15	66	71	0	0	Hatch
192	Day 4 after withdrawal	10	80	100	60	75	Break-out
240	Day 6 after withdrawal	5	80	100	60	75	Break-out
264	Day 7 after withdrawal	5	80	100	67	100	Hatch

Time- indicates the time of eggs collection in relation to treatment. Method- indicates the methodology used to ascertain fertility for the specified time point. Values are presented as the percentage fertility of the total number of eggs incubated. From 120 h, five hens per days were slaughtered for necropsy evaluation

**Fig. 3:**
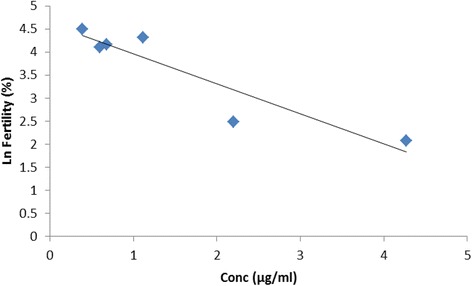
Percentage fertility on the natural logarithmic scale obtained from egg break-out from the batch of eggs incubated (Table 3) versus corresponding egg florfenicol concentration (*n* = 5) from the separate batch of eggs assayed by HPLC

Post-treatment egg concentration for the 90 mg/kg group was 4.27 ± 0.76 μg/g on day 1 and had declined to 0.85 ± 0.05 μg/g by day 5 post-0 mg/kg treatment (Table 4). The depletion was not linear for all the groups with the 30 and 60 mg/kg group showing an increase in concentration from 24 to 48 h, before starting to decline. When the percentage fertility was plotted against florfenicol egg concentrations, a linear relationship was present with linearity of >85 % (Fig. 3). The best fit equation was defined as y =-0.65× + 4.61, and an LC_50_ of 1.07 μg/g.

**Table 4:** Concentration (μg/g) of florfenicol in the eggs (*n* = 5) at 24, 48, 72, 96 and 120 h after the withdrawal of treatment of florfenicol at 30, 60 or 90 mg/kg oid for 5 days

Time after dosing (hour)			Dose
	30 mg/kg	60 mg/kg	90 mg/kg
24	0.68 ± 0.05	2.2 ± 0.30	4.27 ± 0.76
48	1.08 ± 0.11	2.53 ± 0.24	3.8 ± 0.34
72	0.59 ± 0.02	1.8 ± 0.15	1.85 ± 0.28
96	0.39 ± 0.06	0.6 ± 0.04	1.12 ± 0.13
120	0.27 ± 0.01	0.59 ± 0.13	0.85 ± 0.05

## Discussion

This is the first report of an adverse reaction associated with the use of florfenicol in breeding poultry that we are aware of, even though formulations of florfenicol are available for use in broilers internationally. For this study, we exposed an established breeder flock to florfenicol in controlled manner to ascertain if the drug could interfere with fertility as well as to determine if toxicity was at the level of the cockerel, hen and/or embryo. The reason for adopting this specific design came from the non-specific information provided in the spontaneous adverse drug report that related treatment to a subsequent decrease in fertility in a breeder flock. With the treatment being administered in the water and subsequent non-specific exposure of both the cockerels and hens, the resultant toxicity could have been from a direct toxic effect in either the cockerel or hen by interfering with their breeding potential. It is also possible that due to the drug’s lipid solubility, it was distributed into either the albumin or yolk and subsequently was toxic to only the developing embryo.

In general florfenicol was not characterised by any overt signs of toxicity or pathological changes in the treated birds. For phase 1, in which the hens were treated at lower doses of 10, 20 and 30 mg/kg a moderate negative effect on fertility was seen at 20 and 30 mg/kg after four days of consecutive therapy. On egg break-out this was characterised by early embryonic death at approximately day 5 of development. When only the cockerels were treated in phase 2, no changes in fertility were evident. For phase 3, in which the hens were treated at the higher doses of 30, 60 and 90 mg/kg a major, albeit temporary, decrease in fertility characterised as a 100 % decline was evident. As for phase 1, egg break-outs indicated that embryonic mortalities occurred at approximately day 5 of development with good correlation to total egg florfenicol concentrations.

With the absence of overt toxicity in either hens or cockerels, the failure of the product to interfere with cockerel fertility, and the presence of early embryonic death at day 5, it would appear that florfenicol toxicity is limited to the developing embryo as a result of deposition of florfenicol within the egg prior to lay. The effect also appears to be absolute, as in the event that the embryo did not die, normal development to hatch resulted. The concentration response relationship in toxicity also provides a plausible link that the florfenicol was the cause of the embryonic death. This finding does differ to an *in vitro* toxicity study undertaken through the injection of florfenicol at 20 to 30 mg/kg egg weight, into day 4 embryonated eggs from Marandi breed chickens [12]. For the latter study no signs of toxicity was evident at necropsy on 18 day old embryos. While we are uncertain for the difference in sensitivity, breed differences may play a role. Another plausible reason could be the time of exposure, as *in vivo* exposure would result in the zygote being exposed to drug from the point of lay, while the older embryo may have reached a stage of non-susceptibility. As evident in this study, embryos that do survive florfenicol exposure do develop into normal healthy chicks.

What was also evident in the study was the slight difference in the time to maximum concentration (Cmax) within the eggs, with the 90 mg/kg group reaching Cmax at 24 h post-drug withdrawal and the 30 and 60 mg/kg dose reaching Cmax at 48 h. This would suggest that the deposition of florfenicol into the egg is highly variable. This finding is not unusual and can be explained by pharmacokinetic theory which generally describes the deposition of drugs into the egg as being highly individually variable. The reasons for the latter is the need for the drug to distribute into the two media of the egg, namely the albumin and the yolk [13, 14], with the distribution being more rapid into the albumin reaching steady state usually by day 2-3 post-treatment, while steady state in the yolk can take up to 8 days. In the context of this study, the highly variable Cmax evident is explainable by both the variable nature of egg disposition as well as the study design which relied on eggs being randomly sampled per time point. Unfortunately in a natural open breeding system in which the hens and cockerels are both housed on open floors, it is not possible to sequentially collect eggs from the same hen as their eggs are not identifiable.

Despite some previous evidence of embryotoxicity in the mouse, the mechanism of toxicity of florfenicol is yet to be described. Literature is however available for the other molecules in the amphenicol class. In a controlled murine study, 14- or 20-somite embryonic stages exposed to chloramphenicol (the progenitor compound of the amphenicol group), at concentrations of 0, 200, or 300 μg/ml for 22 – 24 h showed defects of the neural tube (failure to close) and the forebrain as well as the inhibition of haemoglobin formation [15]. The study was able to demonstrate that the toxic effect was due to inhibition of protein synthesis as a result of interference with the messenger RNA. In another study, chloramphenicol injected into turkey hatching eggs to eliminate *Mycoplasma meleagridis* at doses of 2.5 and 5 mg also reduced hatchability, with embryo deaths being recorded before day 9 of the incubation period [16]. Finally in a rat model of teratogenicity it was found that the chloramphenicol interfered specifically with mitochondrial protein synthesis by decreasing the production of mitochondrial cytochrome oxidase enzyme (Cytochrome C oxidase is the terminal enzyme in the electron transport chain located on the inner mitochondrial membrane) [17]. With florfenicol and chloramphenicol both sharing the same mechanism of protein synthesis inhibition, it is likely that florfenicol is toxic to the early stages of embryonic development via foetal protein synthesis inhibition.

Another important finding in this study was the lower susceptible of the study bird than that reported for the adverse drug reaction. While the reason for this difference is not known, it is possible that housing conditions, total days in lay, and the presence of the *E. coli* infections add to the general toxicity of the molecule. While it may be argued that the doses in use were excessively high and therefore unlikely to represent field adverse drug reactions, this is unlikely as the model is based on current regulatory best practice in evaluating drug toxicities. In the standard development of a veterinary medicine, it is a requirement for the drug to be tested at a control overdose (target species toxicity) at a minimum of 3x and 5x an overdose, with 10x overdose being the upper end of the scale [18]. The aim of these target species toxicity studies is to elucidate the toxic potential of a molecular in the target species as well to describe its likely adverse reaction from use i.e. these studies use a higher dose to elucidate likely side effects in a small sample size. For this study, birds exposed to a 3x to 9x overdose all showed signs of embryonic toxicity making it likely that this side effect would result under field conditions. Also important to note is that the non-South African recommended dose for the treatment of broiler chickens is 30 mg/kg, the lowest identified toxic dose for this study [19].

An unexpected finding of this study was the absence of decreased egg fertility when only the cockerels were treated, as previous mammalian have indicated the ability for the drug to induce testicular toxicity. From Ando et al testicular toxicity seen in rats was characterized by vacuolated Sertoli cells was most likely as a result of reduced cytochrome activity [20]. With the cytochrome oxidases being important for aerobic sperm production, it would appear that avian Sertoli cells are less sensitive to the effects of the amphenicols. This also highlights that mammalian toxicity studies are not necessary indicative of toxicity in avian species. However, it must be noted that this study was designed to investigate the effects of florfenicol on cockerel breeding potential under commercial conditions at a ratio one cockerel to eight hens, with the result that this study design may not have been sensitive enough to elucidate more insidious testicular/epididymal toxicity. As a result, it would still be important to evaluate the direct effect of florfenicol in a larger group of breeding-aged cockerels, for further sex-related side effects.

## Conclusion

We conclude that florfenicol has the potential to induce early embryonal death in chickens. We would recommend that the drug be avoided in breeding birds, unless the value of the breeder flock dictates that the health of the flock and return to productivity is more important than resultant decrease in egg fertility.
